# Real World Overall Survival of Patients With Metastatic Renal Cell Carcinoma Treated With Only Available Sunitinib and Pazopanib in First-Line Setting

**DOI:** 10.3389/fonc.2022.892156

**Published:** 2022-06-08

**Authors:** Predrag Nikic, Nada Babovic, Zoran Dzamic, Svetlana Salma, Vesna Stojanovic, Suzana Matkovic, Zoran Pejcic, Kristina Juskic, Ivan Soldatovic

**Affiliations:** ^1^Clinic of Urology, University Clinical Center of Serbia, Belgrade, Serbia; ^2^University of Belgrade, Faculty of Medicine, Belgrade, Serbia; ^3^Institute for Oncology and Radiology of Serbia, Belgrade, Serbia; ^4^Oncology Institute of Vojvodina, Sremska Kamenica, Serbia; ^5^University Clinical Center Nis, Clinic of Oncology, Nis, Serbia; ^6^Institute of Medical Statistics and Informatics, Belgrade, Serbia

**Keywords:** metastatic clear cell renal carcinoma (mRCC), overall survival (OS), sunitinib, pazopanib, limited access to cancer therapy, real world data

## Abstract

**Background:**

The emerging new standard of care for metastatic clear cell renal carcinoma (mRCC) becomes a challenge when access to new drugs is limited. In Serbia, sunitinib and pazopanib are the only available first-line therapies. The second-line treatment for mRCC has never been and is still not available. We aimed to assess overall survival (OS) in patients with mRCC who received first-line sunitinib or pazopanib when access to second-line treatment was not available.

**Methods:**

This retrospective observational study analyzed data from a nationally representative cohort of 759 patients who started on first-line sunitinib or pazopanib between 1 January 2012 and 30 June 2019, in 4 centers in Serbia. The data cut-off date was 31 December 2019. Key eligibility criteria were clear cell RCC histology, measurable metastatic disease, performance status 0 or 1, and the Memorial Sloan Kettering Cancer Center favorable or intermediate prognosis. The primary outcome was OS from the start of first-line treatment to death or data cut-off date.

**Results:**

The study population included 759 patients with mRCC who started with first-line sunitinib (n = 673; [88.7%]) or pazopanib (n = 86; [11.3%]). Overall, the mean age was 61.0 ± 9.7 years at treatment baseline, and 547 (72%) were men. mRCC was primarily diagnosed in 230 (30%) patients, and most of them underwent cytoreductive nephrectomy prior to systemic therapy (n = 181 [79%]). Additional treatment of metastases prior to and/or during treatment was used in 169 patients (22.3%). Grade 3 and 4 adverse events occurred in 168 (22.1%) and 47 patients (6.2%), respectively, and treatment was permanently stopped because of toxicity in 41 (6.9%). The OS was calculated from the start of first-line treatment, and the median follow-up was 14 months (range, 0–97). The median OS in the entire cohort was 17 months (95% CI, 14.6–19.4).

**Conclusions:**

With only available sunitinib and pazopanib in first-line treatment, modest improvements are seen in the overall survival of patients with mRCC in real world clinical practice. In circumstances of limited availability of cancer medicines, our results can contribute to accelerating patient access to novel cancer therapies that have been shown to prolong survival in mRCC.

## Introduction

Among all urological malignant tumors, renal cell carcinoma (RCC) is the third most common, after prostate cancer and urothelial cancer (1). As a serious and potentially lethal disease, it presents a challenge in multidisciplinary treatment. Approximately 30% of patients with clear cell RCC are initially diagnosed in the metastatic stage of disease, and 1/3 of patients will relapse with metastases up to 3 years after being surgically treated for initially localized clear cell RCC (2). Systemic targeted therapy and immunotherapy are the mainstays of treatment for metastatic clear cell RCC (mRCC). Owing to emerging evidence from clinical trials demonstrating improved overall survival with both dual immunotherapy and a combination of immunotherapy with targeted therapy compared to single-agent Tyrosine kinase inhibitor (TKI) therapy, international treatment guidelines and recommendations for first-line treatment of mRCC have been dramatically changed in recent years (3, 4). Moreover, real world data published so far on this topic appear to be similar to results found in recently reported clinical trials (5). Second-line therapy is a necessary part of treatment and options are based on the treatment received in the first-line setting. However, following treatment recommendations for mRCC becomes a challenge when access to new drugs is limited. In Serbia, sunitinib and pazopanib are the only available first-line targeted therapies. The second-line treatment for mRCC has never been and is still not available. Limited evidence exists on treatment outcomes of first-line targeted therapy when access to second-line treatment for mRCC is not available. Other studies which explored treatment outcomes of first-line targeted therapy focused more on choosing the appropriate options in second-line treatment rather than on maintaining first-line treatment for as long as possible.

This study is the first retrospective observational study conducted in Serbia with the aim of describing treatment characteristics and survival outcomes in patients with mRCC who received first-line sunitinib or pazopanib in a real-world setting when access to second-line treatment is not available.

## Materials and Methods

### Study Design

This was a national, multicenter, retrospective observational study conducted in Serbia, focused on evaluating first-line TKI treatment survival outcomes in patients with clear cell mRCC in a real-world setting. A total of 759 patients who started on first-line TKI between January 1, 2012, and June 30, 2019, were enrolled in this study. This number represents the national cohort of the entire patient population treated in 4 centers in Serbia. The data cutoff for this analysis was 31 December 2019, and patients were followed-up for at least 6 months after enrollment. Key eligibility criteria were age ≥18 years, histologically confirmed diagnosis of ccRCC, measurable disease according to the Response Evaluation Criteria in Solid Tumors (RECIST v1.1), performance status 0 or 1, and Memorial Sloan Kettering Cancer Center (MSKCC) favorable or intermediate prognosis. All patients who were given at least 1 dose of first-line TKI were eligible for the study. Patients not included in this study represent a population with non-clear cell RCC, performance status ≥2 and MSKCC poor prognosis who cannot receive first-line TKI in Serbia according to administrative rules proposed by the National Health Insurance Fund (NHIF). Patients treated as part of clinical research trials were not included. Data were retrospectively collected from electronic and paper medical records, and included demographic and clinical characteristics at baseline, treatment characteristics, and survival outcome data. In Serbia, sunitinib was available from November 2011, and pazopanib became available in March 2017. Patients received either sunitinib or pazopanib as a first-line regimen, according to the agreed choice of the physician and patient. The sunitinib starting dose was 50 mg once daily, either on the standard sunitinib schedule (4/2 schedule) or alternate dosing schedule (2/1 schedule). Pazopanib was given at a starting dose of 800 mg once per day. Subsequent dose reductions and/or switches to an alternate 2/1 dosing schedule for sunitinib were applied in accordance with personal tolerability. Patients were stratified into favorable and intermediate risk groups according to five MSKCC risk factors. Treatment-related toxicities were graded according to the National Cancer Institute CTCAE v5.0. Safety assessments were based on the occurrence of toxicity grade 3 or 4 during the treatment. The RECIST v.1.1 criteria were used to evaluate treatment response. Radiographic assessment was performed every 3 months during the first year of treatment and then every 6 months. Treatment response was evaluated only for patients who had had at least one radiographic assessment. Duration of treatment was defined as the time from the initiation of treatment to permanent treatment discontinuation for any reason. The definition of disease progression includes radiographic or clinical progression alone or a combination of radiographic and clinical progression. The reasons other than disease progression, death from any cause or adverse event leading to permanent treatment discontinuation included—the patient was lost to follow-up or wished to end treatment. The primary endpoint of the study was the evaluation of overall survival (OS) for first-line TKI therapy. OS was defined as the time from the initiation of first-line TKI therapy to death from any cause. The other outcome of interest was progression-free survival (PFS), defined as the time from the initiation of first-line TKI therapy to the earliest date of physician-assessed disease progression or death from any cause. Patients who were lost to follow-up and those who were still alive or had not progressed at the end of the data cutoff analysis were censored in statistical analysis. The study was approved by the Ethical Committee of the Institute of Oncology and Radiology of Serbia, Belgrade under number 3134/01, issued on 25 August 2020.

### Statistics

Results were presented as frequencies (%), means ± standard deviation or median (interquartile range) depending on data type and distribution. Kaplan–Meier analysis was used to estimate the progression-free survival and overall survival. Survival is presented as the median (95% confidence interval). Statistical analyses were performed using R 4.0.2 (R Core Team, 2017) and conducted from March to July 2021.

## Results

### Demographic and Clinical Characteristics

This observational study included 759 patients with clear cell mRCC who were started with the first-line TKI sunitinib or pazopanib in 4 institutions in Serbia between 1 January 2012 and 30 June 2019. All patients received at least 1 dose of TKI. Demographic and clinical characteristics at baseline are shown in **Table 1**. Most of the patients in the cohort were men (N = 547, 72%). Overall, the mean age was 61.0 ± 9.7 years at the treatment baseline. Primary diagnosis confirmed the localized disease in 529 (70%), and locally advanced or metastatic disease in 230 (30%) patients. Most patients initially diagnosed with metastatic disease have undergone nephrectomy before systemic therapy (N = 181, 79%). According to the MSKCC prognostic criteria, 504 (66%) patients had an intermediate prognosis, with two risk factors found in 351 (70%). Based on available data, a calculation of the IMDC risk score was performed on 146 patients. In this subgroup, according to the MSKCC prognostic criteria, 36 (25%) patients had a favorable prognosis, and 110 (75%) patients had an intermediate prognosis. When recalculating the risk score according to IMDC criteria, 25 (22.7%) patients with the MSKCC intermediate prognosis were reclassified to the IMDC poor prognosis. A median number of 2 metastatic sites were radiographically confirmed at baseline, and the most common were the lungs (73%), lymph nodes (64%), bones (23%), local recurrence (20%), liver (16%), and adrenal gland (14%). According to the inclusion criteria, patients with a poor MSKCC prognosis and/or performance status ≥2 and/or with non-clear cell RCC histology were not candidates for first-line TKI treatment.

**Table 1:** Demographic and clinical characteristics at baseline.

Characteristic	No. (%)	No. (%)	No. (%)
	Total (n = 759)	Sunitinib (n = 673)	Pazopanib (n = 86)
**Gender**			
Male	547 (72.1%)	492 (73.1%)	55 (64.0%)
Female	212 (27.9%)	181 (26.9%)	31 (36%)
**Age at initial diagnosis,** mean ± SD, y	58.7 ± 9.7	58.2 ± 9.6	62.9 ± 9.4
**Age at start of 1L TKI,** mean ± SD, y	61.0 ± 9.7	62.9 ± 9.4	65.3 ± 9.2
**Stage at initial diagnosis**			
Localized	529 (69.7%)	487 (72.5%)	41 (47.7%)
Metastatic	225 (29.6%)	184 (27.4%)	41 (47.7%)
Locally advanced	5 (0.7%)	1 (0.1%)	4 (4.7%)
**Initially diagnosed *Locally advanced/Metastatic RCC***	*n = 230*	*n = 185*	*n = 45*
Nephrectomy prior TKI 1L	181 (78.7%)	152 (82.1%)	29 (64.4%)
Nephrectomy during TKI 1L	12 (5.2%)	9 (4.9%)	3 (6.7%)
Nephrectomy not performed	37 (16.1%)	24 (13%)	13 (28.9%)
**Prognostic MSKCC**			
Favorable	255 (33.6%)	227 (33.7%)	28 (32.6%)
Intermediate	504 (66.4%)	446 (66.3%)	58 (67.4%)
Intermediate-1 risk	153 (30.4%)	138 (30.9%)	15 (25.9%)
Intermediate-2 risk	351 (69.6%)	308 (69.1%)	43 (74.1%)
**Prognostic MSKCC to IMDC**	*n = 110*	*n = 86*	*n = 24*
Intermediate to Favorable	1 (1%)	1 (1.2%)	0
Intermediate to Intermediate	84 (76.3%)	69 (80.2%)	15 (62.5%)
Intermediate to Poor	25 (22.7%)	16 (18.6%)	9 (37.5%)
**Performance status**			
0	570 (75.1%)	509 (75.6%)	61 (70.9%)
1	189 (24.9%)	164 (24.4%)	25 (29.1%)
**MSKCC risk factors present**			
Time <12 months	400 (52.7%)	349 (51.9%)	51 (59.3%)
LDH >1.5 ULN	49 (6.5%)	37 (5.5%)	12 (14.0%)
Hgb < LLN	150 (19.8%)	121 (18.0%)	29 (33.7%)
Corrected Ca >10 mg/dl	28 (3.7%)	22 (3.3%)	6 (7.0%)
**Number of metastatic sites**	2 (2-3)	2 (2-3)	3 (2-3)
**Site of distant metastases**			
Lungs	553 (72.9%)	486 (72.2%)	67 (77.9%)
Liver	120 (15.8%)	110 (16.3%)	10 (11.6%)
LN mediastinum	251 (33.1%)	221 (32.8%)	30 (34.9%)
LN retroperitoneum	194 (25.6%)	175 (26.0%)	19 (22.1%)
LN other	40 (5.3%)	34 (5.1%)	6 (22.1%)
Local recurrence	152 (20.0%)	134 (19.9%)	6 (7.0%)
Adrenal gland	106 (14.0%)	87 (12.9%)	18 (20.9%)
Bones	175 (23.1%)	144 (21.4%)	31 (36.0%)
Pancreas	27 (3.6%)	21 (3.1%)	6 (7.0%)
Peritoneum	23 (3.0%)	20 (3.0%)	3 (3.5%)
Pleura	41 (5.4%)	31 (4.6%)	10 (11.6%)
Brain	32 (4.2%)	27 (4.0%)	5 (5.8%)
Kidney	59 (7.8%)	54 (8.0%)	5 (5.8%)
Spleen	14 (1.8%)	12 (1.8%)	2 (2.3%)
Other	43 (5.7%)	41 (6.1%)	2 (2.3%)

TKI, tyrosine kinase inhibitor; 1L, first-line; RCC, renal cell carcinoma; MSKCC, Memorial Sloan Kettering Cancer Center; LN, lymph nodes.

### Treatment Characteristics

In total, 673 patients (88.7%) received sunitinib as first-line treatment, whereas the remaining 86 patients (11.3%) were administered pazopanib. TKI treatment crossover was not permitted. The characteristics of first-line TKI treatment are shown in **Table 2**. Treatment started either with the standard sunitinib schedule (4/2 schedule) in 515 patients (76.5%) or with an alternate dosing schedule (2/1 schedule) in 158 patients (23.5%). During the treatment, 156 (30%) of patients receiving the standard schedule were switched to an alternate dosing schedule. All patients started with 50 mg of sunitinib or 800 mg of pazopanib, and dose reduction was necessary for 99 patients (13%). Most of the reasons for dose reduction were treatment-related AE. At the data cutoff, 164 patients (21.6%) were still receiving therapy and 595 patients (78.4%) permanently discontinued first-line TKI. The most common reason for permanent discontinuation of first-line TKI was disease progression in 364 patients (61%). In our study, the definition of disease progression included radiographic or clinical progression alone, or a combination of radiographic and clinical progression.

**Table 2:** First-line TKI treatment characteristics.

Characteristic	No. (%)	No. (%)	No. (%)
	Total (n = 759)	Sunitinib (n = 673)	Pazopanib (n = 86)
**Treatment schedule at start of sunitinib**			
4/2		515 (76.5%)	
2/1		158 (23.5%)	
**Treatment schedule switched to2/1 sunitinib**		156 (30.1%)	
**Dose reduction**	99 (13.0%)	88 (13.1%)	11 (12.8%)
**Reason for dose reduction**			
Adverse event		86 (97.7%)	11 (100%)
Other		2 (2.3%)	0
**Sunitinib dose reduction**			
37.5 mg		88 (100%)	–
25 mg		0	–
**Pazopanib dose reduction**			
600 mg		–	9 (81.8%)
400 mg		–	2 (18.2%)
**Permanent treatment stops**	595 (78.4%)	541 (80.4%)	54 (62.8)
**Reason for permanent treatment stop**	*n = 595*	*n = 541*	*n = 54*
Death	145 (24.4%)	134 (24.8%)	11 (20.4%)
Progression	364 (61.2%)	333 (61.5%)	31 (57.4%)
Adverse events	41 (6.9%)	32 (5.9%)	9 (16.7%)
Other	45 (7.5%)	42 (7.8%)	3 (5.5%)
**Total cycles of 1L TKI, median, (IQR)**	8 (3–14)	8 (3–14)	9 (5–15)
**Duration of the 1L TKI, median, (IQR)**	10 (5–21)	*12 (5–21)*	*9 (5–15)*
**Best Treatment response**			
Complete response (CR)	24 (3.2%)	23 (3.4%)	1 (1.2%)
Partial response (PR)	167 (22%)	146 (21.7%)	21 (24.4%)
Objective Response Rate (ORR = CR + PR)	25.2%	25.1%	25.6%
Stable disease (SD)	394 (51.9%)	346 (51.4%)	48 (55.8%)
Progressive disease (PD)	60 (7.9%)	60 (8.9%)	0
Not evaluated	114 (15%)	98 (14.6%)	16 (18.6%)
**Metastases therapy prior and/or during treatment**	169 (22.3%)	148 (22.0%)	21 (24.4%)
Surgery	44 (25.9%)	42 (28.4%)	2 (9.1%)
Radiosurgery stereotactic	22 (12.9%)	18 (12.2%)	4 (18.2%)
Radiotherapy palliative	135 (79.4%)	117 (79.1%)	18 (81.8%)
**Adverse events**			
Grade 3	168 (22.1%)	144 (21.4%)	24 (27.9%)
Grade 4	47 (6.2%)	40 (5.9%)	7 (8.1%)

TKI, tyrosine kinase inhibitor; 1L, first-line; IQR, interquartile range.

During first-line TKI treatment, stable disease was the most frequently observed best treatment response in 394 patients (51.9%). Radiographic progression at the first evaluation was found in 60 patients (7.9%). Treatment response was not evaluated in 114 patients (15%) because of reasons leading to early discontinuation of first-line TKI. The overall response rate for the entire cohort was 25.2%. Additional treatment of metastases before and/or during first-line TKI was used in 169 patients (22.3%). Permanent treatment discontinuation because of toxicity occurred in 41 patients (6.9%). Grade 3 AEs were recorded in 168 patients (22.1%) and Grade 4 AEs in 47 patients (6.2%).

### Survival Outcomes—PFS and OS

**Figure 1:**
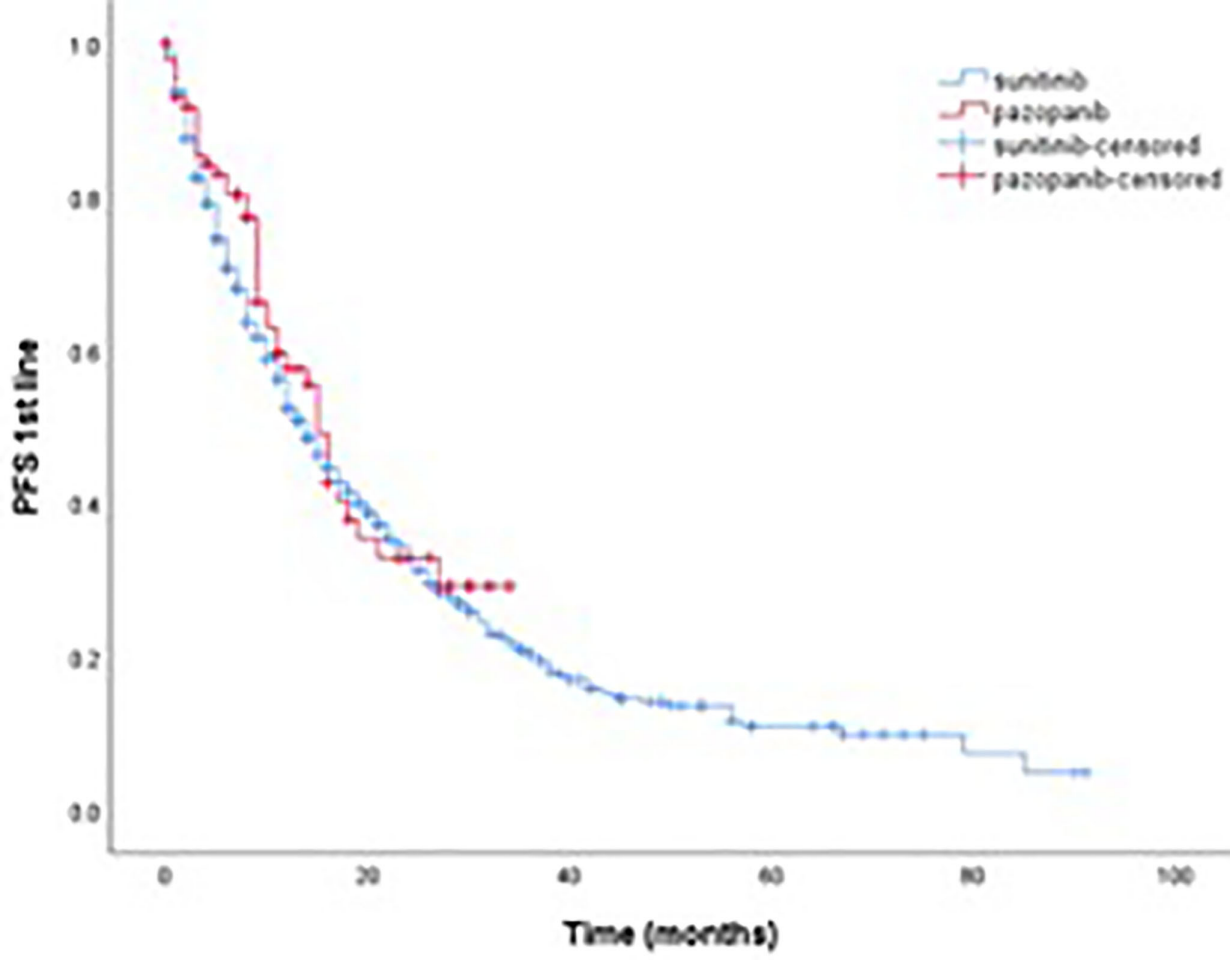
Kaplan–meier estimate of PFS for first-line sunitinib and pazopanib.

**Figure 2:**
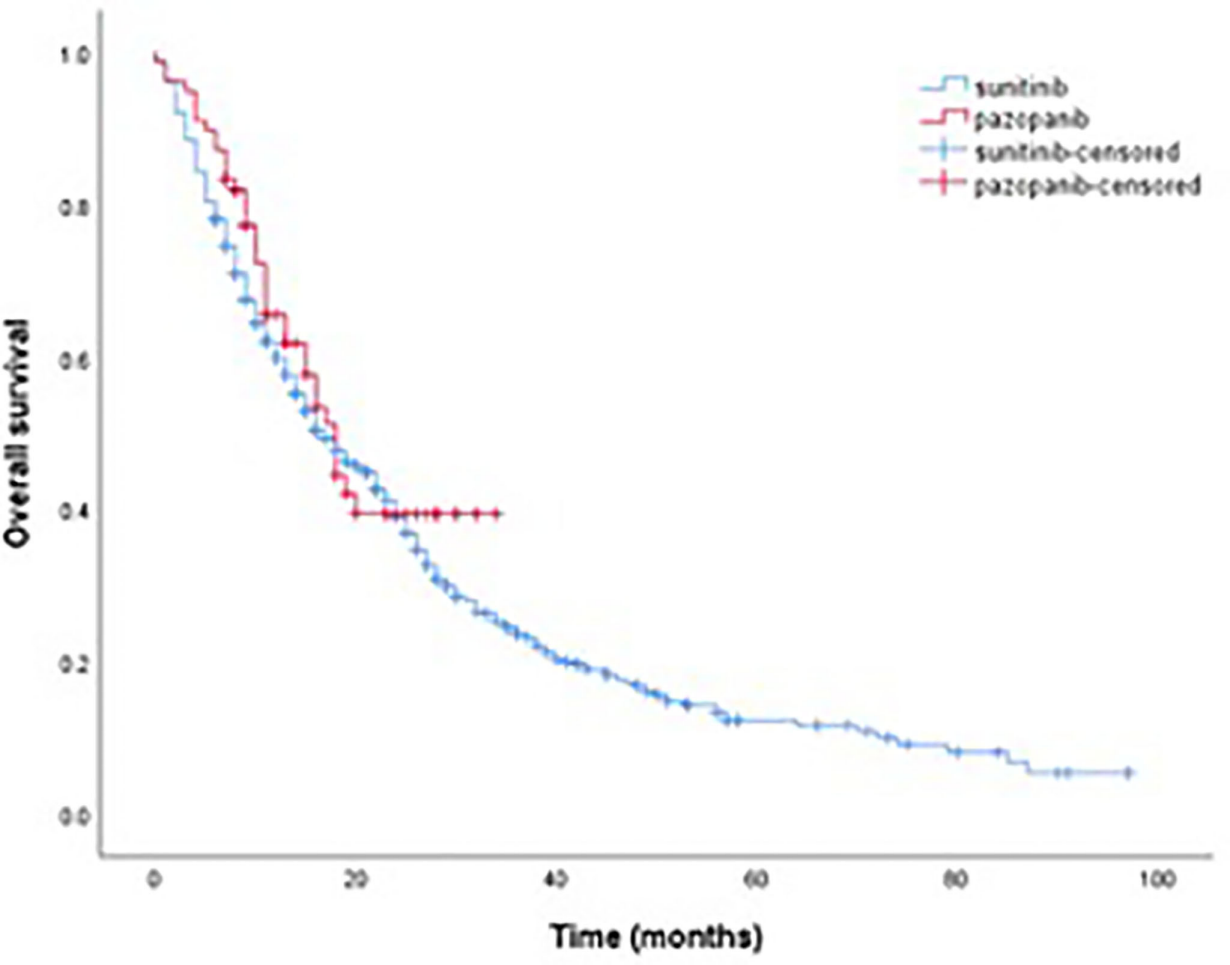
Kaplan–meier estimate of OS for first-line sunitinib and pazopanib.

Among the 759 patients included in the study, 496 (65.4%) had died, 55 (7.2%) were lost to follow-up, and 208 (27.4%) were still alive at the data cut-off. OS was calculated from the start of first-line TKI treatment, and the median follow-up time was 14 months (range, 0–97). Survival outcomes for first-line TKI are shown in **Table 3**. The median PFS and median OS in the sunitinib group were 14 months (95% CI, 12.3–15.7) and 17 months (95% CI, 14.2–19.8), respectively. The median PFS and median OS in the pazopanib group were 15 months (95% CI, 13.0–16.9) and 18 months (95% CI, 14.9–21.0), respectively. Median PFS and median OS in the entire cohort were 14 months (95% CI, 12.4–15.6) and 17 months (95% CI, 14.6–19.4), respectively. Kaplan–Meier estimates of PFS and OS for first-line sunitinib and pazopanib are shown in **Figures 1**, **2**, respectively.

**Table 3:** Survival outcomes of First-line TKI treatment.

Survival outcomes	Median (95% CI)	Median (95% CI)	Median (95% CI)
	Total n = 759	Sunitinib n = 673	Pazopanib n = 86
PFS 1L	14 (12.4–15.6)	14 (12.3–15.7)	15 (13.0–16.9)
OS 1L	17 (14.6–19.4)	17 (14.2–19.8)	18 (14.9–21.0)

TKI, tyrosine kinase inhibitor; 1L, first line; PFS, progression free survival; OS, overall survival; CI, confidence interval.

## Discussion

For the most part, when access to a subsequent line of treatment for treating patients with clear cell mRCC is still lacking, clinicians in Serbia are challenged with how to maximize clinical effectiveness and how to maintain first-line TKI treatment for as long as possible.

In this observational study, we analyzed data collected retrospectively from the medical records of 759 patients with clear cell mRCC who received sunitinib or pazopanib in first-line treatment. Our goal was to present the characteristics and survival outcomes of treatment in everyday clinical practice when second-line therapy is not available.

The general characteristics of our patients are in accordance with published data from the literature (6–9). At the time of initial diagnosis, around 30% of patients in our study population had metastatic RCC, which is in line with previously reported data (2). The role of cytoreductive nephrectomy and metastasectomy in patients with mRCC has been controversial to date. According to data published so far from two randomized and a number of retrospective studies, the use of surgical treatment before and/or during systemic therapy in patients with mRCC should be carefully considered in accordance with patient characteristics and disease (10). Therefore, it is not surprising that in 79% of our patients primarily diagnosed with mRCC, cytoreductive nephrectomy was performed before the start of targeted therapy. However, even with the goal of maintaining first-line TKI as long as possible, the addition of metastasectomy and stereotactic radiosurgery was less used before and/or during systemic therapy in our patient population, mainly due to a lack of evidence-based data. It is known that in clinical practice, optimizing the dosing regimen provides an improvement in the efficacy and safety of the therapy. Keeping in mind that first-line TKI is the only available treatment for patients with mRCC in Serbia, it was decided to avoid unnecessary treatment discontinuation due to adverse events. All patients in our study started treatment with the standard dose of sunitinib or pazopanib, which differs from the reported 75–87% in observational studies (11–19). In our cohort, 23% of patients started with a 2/1 sunitinib schedule, and 30% of patients who started with a 4/2 regimen were switched to a 2/1 dosing schedule. Only 13% of patients in both treatment groups had a dose reduction during treatment, which is significantly less compared to data reported in the COMPARZ study (pazopanib 44% and sunitinib 51%) (8), but is also less in comparison with studies from everyday practice (21–67%) (11–19). Findings published so far have shown that sunitinib was better tolerated in patients who received a 2/1 regimen compared with those on a standard 4/2 regimen (20), with similar treatment outcomes (21).

During the treatment, grade 3 and 4 AEs occurred in 28% of patients in our study, which is lower compared to data from the literature. To date, a very wide range of reported grade 3 and 4 AEs can be found in published phase 3 studies. Moreover, in comparing different phase 3 clinical studies, great variability in the occurrence of grade 3 and 4 AEs becomes obvious even with the same drug. Namely, in the phase 3 registration study for pazopanib, 33% of grade 3 and 4 AEs were reported (7), while 74% of patients in the COMPARZ study had grade 3 and 4 toxicities with pazopanib and sunitinib (8). However, data collected retrospectively together with less strict reporting of AE in everyday practice contributes to the variable results reported in observational studies. Namely, the significantly lower rate of grade 3 and 4 AEs reported in everyday practice may indicate that their actual occurrence is underestimated (11–19). However, it should also be noted that most TKI-related AE can be prevented and successfully treated when recognized in time (22). In our study, only 7% of patients permanently discontinued treatment because of significant TKI toxicity.

This study aimed to assess the survival outcomes of patients with mRCC who received sunitinib or pazopanib as first-line treatment in everyday practice when subsequent therapy was not available. According to data published so far in the literature, both in phase 3 studies (6–8) and observational studies (11–19), the most common reason for permanent discontinuation of first-line TKI was disease progression. The median PFS in our patients was 14 months, which is very similar to the results of registration studies both for sunitinib and pazopanib (11 months) in the first-line of mRCC treatment (6, 7). However, in retrospective studies from everyday practice, the median PFS for first-line TKI ranged from 8.4 to 23.8 months (11–19). Huge variations in mPFS observed in retrospective studies can be explained partly by differences in radiographic assessment intervals in routine clinical practice. Moreover, note that PFS is mostly based on the subjective assessment of researchers. This is notably evident in real-world evidence, where most of the criteria for disease assessment are not clearly defined. Nevertheless, in Serbia, where patients cannot continue with the subsequent line of treatment, radiographic progression is assessed more carefully to avoid mistakes and unnecessary treatment cessation. The primary endpoint of this study was the evaluation of OS in patients treated for mRCC with the only available sunitinib or pazopanib in a first-line setting.

Our study showed that patients with clear cell mRCC treated with sunitinib or pazopanib in first line had worse OS (17 months; CI 95% 14.6–19.4) compared with the results of phase 3 studies in the same population. A comparative study (COMPARZ) showed that pazopanib and sunitinib had similar OS (28.4 vs. 29.3 months; HR 0.91; CI 95% 0.79–1.06; p = 0.275) (8). Given the relatively similar demographic and clinical characteristics of the patient population in our study and the phase 3 studies, the estimated OS should be expected to be comparable. However, OS reported in clinical practice studies varies widely between 16.7 and 44 months (11–19), which reflects the presence of significant heterogeneity in clinical characteristics of patients and differences in managing therapy compared to the strict protocol used in phase 3 studies. In addition, our findings are consistent with observational studies examining the OS in patients receiving sunitinib or pazopanib in first line, according to the MSKCC or IMDC risk score (23, 24). Namely, patients with intermediate prognosis were divided into those who have one, and those who have two risk factors in MSKCC or IMDC risk score. The results of these studies showed that OS in patients who had intermediate-1 risk was more similar to OS in patients who had favorable prognosis according to MSKCC or IMDC criteria. In contrast, OS in patients who had intermediate-2 risk was more similar to OS in patients who had poor prognosis according to MSKCC or IMDC criteria. Rini et al. (23) showed that for first-line sunitinib, the median OS for patients with MSKCC intermediate-1 risk was 23.1 months and 16.7 months for patients with intermediate-2 risk. Similar results were reported for first-line sunitinib by Schmidinger et al. (24) with a median OS of 20.5 months and 15.1 months in patients with intermediate IMDC prognosis who had one or two risk factors, respectively. In our study, according to MSKCC criteria, 504 (66%) patients had an intermediate prognosis, with two risk factors found in 351 (70%). It is likely that the population of patients at intermediate risk has the greatest impact on OS in our cohort.

In Serbia, the second-line therapy for mRCC has never been and is still unavailable. The number of patients who are candidates for continuing treatment in Serbia is 35–40%. According to available data from the literature, the number of patients who continue treatment after the failure of the first-line TKI ranges between 26 and 63% in everyday practice (25).

### Limitations

The limitations of this study are mostly marked by its retrospective nature. In addition, to comply with strict administrative criteria, only MSKCC but not IMDC risk score was used in selecting patients to start with treatment, which makes comparison with other retrospective studies difficult. Furthermore, combination therapy has become the new standard of care for the first-line treatment of mRCC, making the data on single-agent TKI treatment less relevant. However, these limitations simultaneously reflect everyday clinical practice, which is also the quality of this study.

## Conclusion

In this representative national cohort study, with only available sunitinib and pazopanib as first-line treatments, modest improvements are seen in the overall survival of patients with mRCC in real-world clinical practice. When access to a subsequent line of treatment and new cancer medicines is limited, common sense and clinical experience become more obvious than strictly following the treatment guidelines that cannot be followed. Considering the emerging new standard of care for clear cell mRCC, our results can accelerate patient access to novel cancer therapies shown to prolong survival in mRCC.

## Data Availability Statement

The original contributions presented in the study are included in the article/supplementary material. Further inquiries can be directed to the corresponding author.

## Ethics Statement

The study was approved by the Ethical Committee of Institute of Oncology and Radiology of Serbia, Belgrade under the number 3134/01 issued on 25 August 2020. Written informed consent for participation was not required for this study in accordance with the national legislation and the institutional requirements.

## Author Contributions

PN and NB contributed to conception and design of the study. PN, NB, ZD, SS, VS, SM, ZP, KJ, and IS contributed to the acquisition, analysis, or interpretation of data. PN and SM wrote first draft of the manuscript. PN and IS performed statistical analysis. All authors listed have made a substantial, direct, and intellectual contribution to the work and approved it for publication.

## Funding

Financial support for open access publication fees will be received by the Serbian Society of Medical Oncology (UMOS) with Independent General Research Grant Number 57567877 provided by Pfizer SRB d.o.o.

## Conflict of Interest

PN, NB, ZD, SS, VS, and SM received honoraria for lectures and travel grants from Pfizer and Novartis outside the submitted work. No other disclosures were reported.

The remaining authors declare that the research was conducted in the absence of any commercial or financial relationships that could be construed as a potential conflict of interest.

## Publisher’s Note

All claims expressed in this article are solely those of the authors and do not necessarily represent those of their affiliated organizations, or those of the publisher, the editors and the reviewers. Any product that may be evaluated in this article, or claim that may be made by its manufacturer, is not guaranteed or endorsed by the publisher.
